# Autoimmune Encephalitis With Positive Anti-Leucine-Rich Glioma-Inactivated 1 (Anti-LGI1) Antibody Mimicking Psychogenic Non-epileptic Seizures

**DOI:** 10.7759/cureus.94129

**Published:** 2025-10-08

**Authors:** Ahmer A Longi, Misbah Fazlani, Sidra Akram, Kasid Nouri, Pushparaja Shetty

**Affiliations:** 1 Internal Medicine, Mediclinic Welcare Hospital, Dubai, ARE; 2 Neurology, Mediclinic Welcare Hospital, Dubai, ARE

**Keywords:** anti-lgi1 antibody, autoimmune encephalitis, psychogenic non-epileptic seizures (pnes), rituximab (rtx), tonic-clonic seizure

## Abstract

Autoimmune encephalitis (AIE) represents a significant cause of neurological and psychiatric disorders, often initially misdiagnosed due to a diverse clinical presentation. This case describes a middle-aged woman initially diagnosed with psychogenic non-epileptic seizures (PNES) following emotional stress and depressive symptoms after a family bereavement. Progressive involuntary jerky movements, cognitive dysfunction, and a generalized tonic-clonic seizure raised suspicion for AIE. Diagnostic imaging showed medial temporal lobe hyperintensities, and serological testing confirmed high titers of anti-leucine-rich glioma-inactivated 1 (LGI1) antibodies. The patient showed significant improvement after receiving pulse steroid therapy and intravenous immunoglobulin (IVIG). This case highlights the importance of recognizing psychiatric and neurological overlap in anti-LGI1 encephalitis, emphasizing the need for prompt diagnostic evaluation and early immunomodulatory treatment to optimize patient outcomes.

## Introduction

Autoimmune encephalitis (AIE) is the third most common cause of encephalitis, with a prevalence rate of 13.7 cases per 100,000 [1]. It was conventionally associated with antibodies triggered by neoplasms, but in recent years, a new group has been recognized as not associated with malignancy [2]. It can be related to different neural-specific autoantigens, of which antibodies against voltage-gated potassium channels (VKGC) are most common [3]. It can further be divided into three types, each targeting different parts of VKGC. Anti-leucine-rich glioma-inactivated 1 (LGI1) encephalitis is typically found in individuals over 60 years of age. It can clinically present as limbic encephalitis (LE) [4]. Encephalitis related to LGI1 is more common. AIE is a rapid neurological disorder characterized by inflammation of the nervous system caused by an abnormal immune response leading to the production of onconeural antibodies [1]. Mental illnesses are divided into idiopathic and secondary forms with identifiable organic causes.

AIE can cause neuropsychiatric syndromes, leading to epileptic seizures or movement disorders with psychopathological anomalies, such as hallucinations, depressive moods, or cognitive defects [5]. Alternatively, some LE cases may present with progressive dementia and behavioral symptoms [6]. Although the underlying cause of anti-LGI1 encephalitis can include a paraneoplastic syndrome, it is mostly unrelated to tumors [3]. Many of these patients are initially diagnosed with non-organic psychogenic disorders until they present with a confirmed tonic-clonic seizure [7]. Here, we report a patient who was initially suspected of having a somatic symptom disorder due to psychogenic non-epileptic seizures (PNES), which was later confirmed to be a part of anti-LGI1 LE. After immunomodulation therapy, the patient had a dramatic improvement in cognitive functioning and seizure control.

## Case presentation

A woman in her 40s, known to have hypothyroidism and hypertension, sought a second opinion at our medical facility due to worsening jerky movements of her limbs. According to her, she was doing well a couple of months back, when she developed low mood and depressive symptoms that began shortly after the death of a close family member. She was managed conservatively with antidepressant medications. Approximately one month later, she began experiencing involuntary jerky movements involving her left upper and lower limbs. These jerky movements initially occurred once or twice daily but progressively increased to 20 to 30 episodes per day over a span of three to four days. These episodes were exacerbated by emotional stress but were not associated with loss of consciousness.

**Figure 1:**
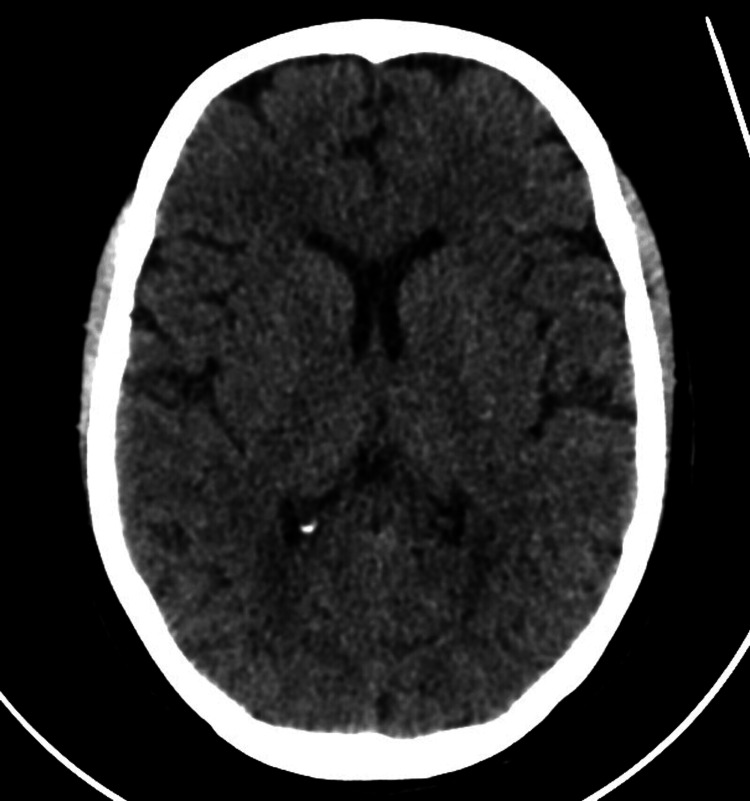
CT head axial view with no new intracranial bleed or space-occupying lesion.

**Figure 2:**
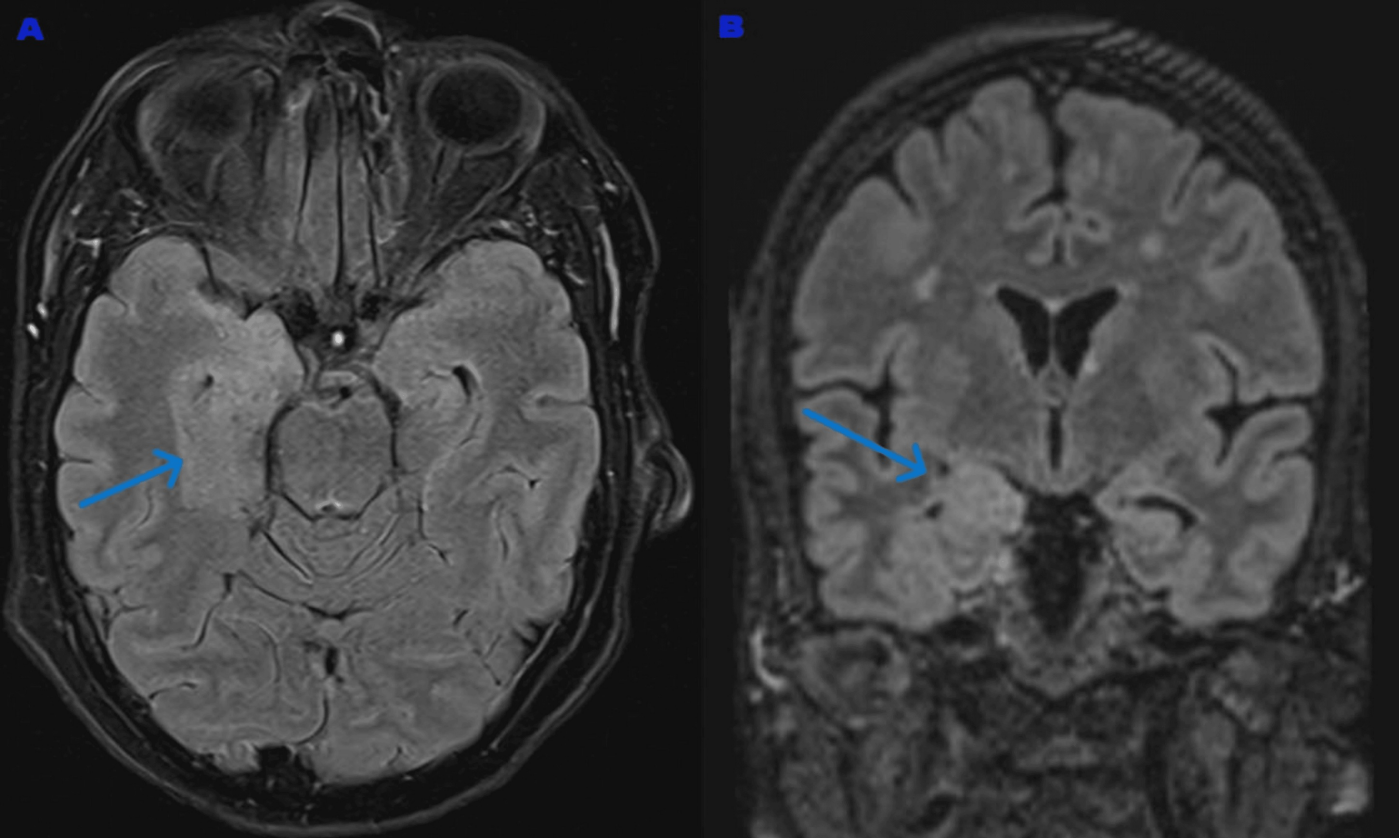
(A) Axial FLAIR MRI of the brain demonstrating right medial temporal lobe hyperintensity (blue arrow). (B) Coronal FLAIR MRI of the brain demonstrating right medial temporal lobe hyperintensity with edema and loss of fissure (blue arrow). MRI: magnetic resonance imaging; FLAIR: fluid-attenuated inversion recovery

She experienced an episode of a generalized tonic-clonic (GTC) seizure lasting approximately five minutes, which was witnessed by her son. She was asleep when the episode started. It involved oral secretions, but there was no tongue bite or incontinence. However, post-ictal confusion and amnesia followed the event and lasted about 30-40 minutes. She was taken to the emergency department of a nearby hospital, where the episode was managed conservatively. She was not started on any antiepileptic medication. During her visit, she underwent a CT head (Figure 1), which was reported as being normal. An electroencephalogram (EEG) was also performed, which was unremarkable. Based on her given history and recent loss, her working diagnosis was functional disorder, involuntary jerks, or a single isolated nocturnal convulsive seizure. A magnetic resonance imaging (MRI) brain (Figure 2) was done, which showed white matter hyperintensities with cortical thickening in the right medial temporal lobe.

Due to the persistence of her symptoms of jerky movements with additional symptoms of anxiety and personality changes, she sought a second opinion at our hospital with one of the neurologists. During consultation, she continued to have jerky movements during the physical exam and, therefore, was advised to get admitted for further workup through the emergency department. Meanwhile, she was started on levetiracetam 500 mg twice daily.

Examination

Her vitals in the emergency department were as follows: blood pressure 126/72 mmHg, heart rate 80 beats/min, respiratory rate 16 breaths/min, and temperature 36.8°C. Neurological exam revealed intermittent, slow, involuntary jerky movements of the left upper and lower limbs, along with involvement of the right upper limb. Higher mental functions were intact. She demonstrated normal power, reflexes, and sensations in both the upper and lower limbs. There were no cerebellar signs, and the gait was found to be normal, along with no signs of pronator drift or extensor plantar response. The cranial nerve exam was within normal limits. There were no meningeal signs.

Investigations

Routine and targeted laboratory investigations were performed (Table 1). A lumbar puncture was also done, which ruled out any signs of infection. Additional blood tests included an AIE panel, which was positive for anti-LGI1 encephalitis with a titer of 1:1,000. It is a part of the voltage-gated potassium complex associated with LE. Features supporting the diagnosis of autoimmune (LGI1 positive) encephalitis included myoclonus, seizure with amnesia, hyponatremia, mood/personality changes, and temporal lobe hyperintensity on MRI scan.

**Table 1:** Laboratory investigation. LGI: leucine-glioma; VGKC: voltage-gated potassium channel; ALT: alanine aminotransferase; AST: aspartate aminotransferase; CRP: C-reactive protein; HB: hemoglobin; WBC: white blood cell count; PT/INR: prothrombin time/international normalized ratio; IgA: immunoglobulin A; TB PCR: tuberculosis polymerase chain reaction

Test	Result	Unit	Reference range
Anti-LGI1 (VGKC)	Positive (1:1,000)	—	Negative
ALT	12.2	U/L	<45 U/L
AST	18.7	U/L	<35 U/L
Creatinine	51	µmol/L	60–110 µmol/L
CRP	0.8	mg/L	<5 mg/L
HB	10.9	g/dL	F: 12–16, M: 13.5–17.5
WBC	10.7	x10⁹/L	4.0–11.0 x 10⁹/L
Platelets	378	x10⁹/L	150–400 x 10⁹/L
PT/INR	13.5/0.91	sec/ratio	PT: ~11–13.5 sec, INR: 0.8–1.2
IgA	3.20	g/L	0.7–4.0 g/L
Brain 14-3-3	Negative	—	Negative
TB PCR	Negative	—	Negative
Meningitis panel	Negative	—	Negative
Anti-LGI1 (VGKC) repeated in 5 days	Positive (1:100)	—	Negative

Diagnosis and management

Till then, she was being treated for a functional neurological disorder, but based on her history of cognitive symptoms and the presence of involuntary jerky movements, along with MRI findings, a diagnosis of AIE was being considered. With the confirmation of diagnosis, further tests were planned to rule out any paraneoplastic syndromes. She was started on a pulse steroid dose of methylprednisolone 1 g once daily for a total of four days. This was complemented with intravenous immunoglobulin (IVIG) administration at a dose of 2 mg/kg for 4-5 days. Levetiracetam was continued for seizure control. She showed gradual improvement of symptoms, including reduced myoclonus, with improved mood. No further seizure episodes were noticed. The patient was discharged on levetiracetam with further treatment planned with two doses of rituximab two weeks apart to prevent any relapse. EEG, MRI brain, and cognitive/mood tracking were initiated. She was restricted from driving for a duration of six months.

Discharge and follow-up

The patient was discharged with complete resolution of her symptoms. On follow-up done in four weeks, she was still having mild symptoms, with the anti-LGI1 titers still being positive. It was decided to start the patient on the biologic agent rituximab, of which she has received two doses two weeks apart. Her symptoms were much better controlled following the administration of rituximab. However, in one month, she again presented to the emergency department with four episodes of GTC seizures with intermittent absence seizures. She was given an intravenous loading dose of levetiracetam along with a new antiepileptic, lacosamide. Her MRI brain scan showed no difference from the last one. Her EEG showed intermittent right temporal sharp wave discharges. She responded well to the addition of lacosamide, and the anti-LGI1 titers were not high (anti-LGI1 titer 1:10). Due to the titers being low, she was not administered any immunosuppressive biologic medication. Her seizures responded well to the voltage-gated sodium channel antagonist lacosamide. She was discharged on dual antiepileptics. In addition, she was advised to complete the paraneoplastic workup, including tumor markers, which were done in another hospital. On further follow-up in one month, she remained seizure-free.

## Discussion

According to the Brighton Collaboration Encephalitis Working Group, the term encephalitis refers to encephalopathy or any other neurological symptoms emerging from brain parenchymal inflammation. The immunological bases of this inflammation were first mentioned by Corsellis et al. in 1968, referring to LE [1]. LE is an inflammatory process of the limbic structures, with various polymorphic clinical symptoms and signs, caused either by paraneoplastic or non-paraneoplastic conditions [8]. Men are affected more than women by AIE [5]; however, according to another study by Alshutaihi et al. [1], AIE and its mimic can affect both sexes equally, with a median age of onset being 61.7 ± 15.5 years. AIE is a rare disease with an incidence of three to nine per million but includes a high rate of misdiagnosed cases [1]. In terms of antibody types found overall, anti-N-methyl-D-aspartate receptor (NMDA-R), antithyroid (thyroid peroxidase), and anti-VGKC-complex antibodies are frequently encountered with AIE screening tests [5].

We describe a case of a middle-aged female patient with a subacute onset of neuropsychiatric symptoms, which were initially treated as PNES till she worsened clinically. Patients’ previous clinical encounters and the temporal relationship between the initiation of symptoms and her emotional stressor led to her being treated for PNES at an earlier stage of her disease. The addition of cognitive symptoms leads to suspicion of the presence of an AIE. The combination of psycho-behavioral or memory problems, speech dysfunction, orofacial-limb dyskinesias, and epileptic seizures, along with radiological changes on MRI brain scan and a highly positive anti-LGI1 titer, enabled us to make a comprehensive diagnosis of anti-LGI1 AIE.

LGI1-associated encephalitis mostly occurs in middle-aged and older adults, with a median age of around 63 years and a male-to-female ratio of 2:1 [3]. In our case, the affected individual was a middle-aged woman. Anti-LGI1 autoimmune encephalitis is associated with amnesia, behavioral and psychiatric disturbances, and epileptic seizures, along with hyponatremia and, in minor cases, autonomic dysfunction [2]. Also, studies show that an accompanying hyponatremia/syndrome of inappropriate antidiuretic hormone secretion (SIADH) was a useful clue and occurred in approximately half the patients [7]. Hyponatremia is a hallmark of anti-LGI1 encephalitis and is reported in 60% of patients with anti-LGI1 AIE [4]. Anti-LGI1 encephalitis presented with seizures, especially faciobrachial dystonic seizures (FBDS), with supportive tests for the condition showing mild to moderate hyponatremia, normal CSF results, or slightly increased cell counts with a unilateral or bilateral hyperintensity in the medial temporal lobes in MRI brain, along with slowing or epileptic discharges in EEG [8]. It is also known that anti-LGI1 antibody positivity can cause insomnia, and disruption of the LGI1 protein can trigger temporal seizures [4].

The initial stages of AIE are marked by somatic symptoms or symptoms that can mimic PNES, often linked to psychological factors or stress [9]. Based on the history provided by the family and the patient herself, including memory problems, changes in her personality, FBDS, and epileptic fits, there is a suspicion that earlier PNES-mimicking episodes could be due to AIE. Clinical features that increase the likelihood of AIE, including subacute onset of dementia-like features and rapid progression thereafter, were present in our patient [6]. The red flags for underlying AIE in this case were polymorphic psychiatric symptoms with cognitive deficits [4]. The VKGC autoimmunity is frequently associated with epileptic seizure, up to 80% have it, and up to 33% of cases have neoplasia as the underlying cause [10]. MRI remains an important modality in the evaluation of brain parenchymal disorders. Patients with VGKC-complex (anti-LGI1) encephalopathy frequently have MRI T2 hyperintensities in mesial temporal lobes [7].

## Conclusions

This case illustrates the complexity and diagnostic challenges associated with AIE, particularly anti-LGI1 encephalitis, due to its overlap with psychiatric disorders such as PNES. Early recognition of characteristic clinical signs such as FBDS, cognitive decline, mood alterations, hyponatremia, and specific imaging findings is crucial. Timely diagnosis and intervention with immunotherapy significantly improve clinical outcomes, underscoring the importance of increased clinical awareness and comprehensive diagnostic approaches for atypical presentations.
